# Data-Driven Soft Sensing for Raw Milk Ethanol Stability Prediction

**DOI:** 10.3390/s26030903

**Published:** 2026-01-30

**Authors:** Song Shen, Xiaodong Song, Haohan Ding, Xiaohui Cui, Zhenqi Xie, Huadi Huang, Guanjun Dong

**Affiliations:** 1 School of Artificial Intelligence and Computer Science, Jiangnan University, Wuxi 214122, China; shensong@stu.jiangnan.edu.cn (S.S.); 6243110028@stu.jiangnan.edu.cn (H.H.); 2 Key Laboratory of Dairy Quality Digital Intelligence Monitoring Technology, Hohhot 011517, China; songxiaodong@mengniu.cn (X.S.); dongguanjun@mengniu.cn (G.D.); 3 Science Center for Future Foods, Jiangnan University, Wuxi 214122, China; 4 School of Cyber Science and Engineering, Wuhan University, Wuhan 430079, China; 5 Department of Chemical & Materials Engineering, University of Auckland, Auckland 1010, New Zealand; xiezhenqi0804@gmail.com

**Keywords:** ethanol stability, soft sensing, raw milk, diffusion model, unbalanced data

## Abstract

Ethanol stability is an important indicator for evaluating the quality and heat-processing suitability of raw milk. Traditional ethanol stability testing relies on destructive laboratory procedures, which are not suitable for large-scale industrial use. In contrast, parameters such as protein, fat, lactose and other basic compositional indicators are already measured routinely in dairy plants through sensor-based or spectroscopic systems. This provides the basis for developing a non-destructive soft sensing approach for ethanol stability. In this study, a soft sensing model was developed to predict ethanol stability from commonly monitored raw-milk intake indicators. An autoencoder was used to examine feature correlations and select variables with stronger relevance to ethanol stability. TabNet was then applied to build the classification model, and a TabDDPM-based data generation method was introduced to address class imbalance in the dataset. The proposed model was trained and tested using three years of industrial raw-milk intake data from a dairy company. It achieved an accuracy of 92.57% and a recall of 90.26% for identifying ethanol-unstable samples. These results demonstrate the model’s strong potential for practical engineering applications in real-world dairy quality monitoring.

## 1. Introduction

Ethanol stability, as a key indicator of dairy quality, characterizes the resistance of milk protein colloids to ethanol-induced coagulation and is widely used to assess the freshness of raw milk and the structural integrity of casein micelles in processed dairy products. The underlying mechanism arises from the ethanol-induced reduction of the dielectric constant of the medium, which promotes the desolvation of casein micelles and subsequently triggers protein aggregation and precipitation. This process is influenced not only by ethanol concentration but is also closely regulated by the intrinsic pH of milk [1]. With increasing acidity (i.e., decreasing pH), the rising hydrogen ion concentration weakens the charge-shielding layer of casein on the micelle surface, thereby diminishing electrostatic repulsion and facilitating aggregation, which markedly reduces ethanol stability. Conversely, as pH increases, the electrostatic repulsion between micelles is strengthened, the ethanol-induced aggregation tendency is attenuated, and the ethanol stability of dairy products correspondingly improves [2,3].

Due to its easy operation and intuitive results, ethanol stability is widely used in industrial dairy production, not only for the rapid identification of raw milk whether there is colostrum adulteration, rancidity and spoilage or pathogenic contamination and other risks, but also as a quality control parameter in pasteurisation, ultra-high-temperature sterilisation, whey protein concentration, and other processing processes, used to predict the protein stability of the dairy heat treatment process [4]. If the ethanol stability of raw milk is insufficient, protein denaturation and aggregation are very likely to occur during high-temperature treatment, eventually forming precipitation or flocculation, which will seriously affect the appearance and flavour of the product, and even lead to the waste of the finished product [5].

The conventional ethanol stability detection methodology primarily involves mixing raw milk with an ethanol aqueous solution at a 1:1 ratio under controlled conditions 20 °C. This mixture is subsequently allowed to stand undisturbed, followed by visual inspection for flocculation or precipitation formation. A systematic approach utilizing an incremental concentration gradient ethanol solution system (e.g., 60%, 65%, 70%, 72%, 74%, 76%, 78%, 80%, 82%, 84%, 86% *w*/*v*) is typically employed to determine the stability profile of samples across varying ethanol concentrations. The ethanol stability point (ES), defined as the maximum ethanol concentration at which no visible coagulation occurs [6], serves as a quantitative indicator for evaluating milk protein stability. In industrial quality control protocols, a predetermined ethanol concentration threshold is established as the critical determinant. Milk batches demonstrating absence of flocculation upon mixing are classified as “ethanol-stable”, whereas those exhibiting visible aggregates are designated as “ethanol-unstable”. Although these procedures are well established, they remain inherently destructive and require a substantial amount of raw milk for each test. Their limited throughput and reliance on manual observation further restrict their suitability for modern processing environments, where continuous and rapid evaluation is increasingly essential. As a result, developing an ethanol stability assessment method that reduces sample consumption while enabling reliable, non-destructive prediction has become an important technical challenge for the dairy industry.

Non-destructive sensing technologies have already proven effective in other aspects of dairy analysis. For example, ISO 9622:2013|IDF 141:2013 [7] specifies the use of mid-infrared absorption spectroscopy for determining fat, protein, lactose, total solids, and solids-non-fat in raw and processed milk as well as cream. Electrical-impedance-based biosensors have also been proposed for rapid estimation of total bacterial counts in raw milk [8]. In parallel with advances in sensing hardware, the recent growth of artificial intelligence has further expanded the methodological landscape for food quality assessment. Deep learning models, in particular, have attracted increasing attention due to their ability to autonomously extract high-level features and model complex nonlinear relationships. Compared with traditional linear modelling approaches [9] and shallow machine-learning algorithms, deep neural networks generally achieve improved predictive accuracy and enhanced generalisation [10]. Together, these developments provide a practical foundation for indirect, data-driven assessment of quality attributes that are otherwise difficult to measure directly.

Within this context, the term soft sensing is used in a pragmatic and application-oriented sense. Specifically, it refers to an indirect estimation approach in which ethanol stability—a property typically assessed through destructive laboratory testing—is inferred from routinely measured physicochemical indicators using a data-driven model. The proposed approach does not constitute a fully integrated real-time process control sensor; rather, it functions as a predictive screening and decision-support tool that operates on existing measurement data to assist industrial quality assessment.

In the dairy industry, raw milk routinely undergoes comprehensive physicochemical analyses to determine key quality parameters including fat content, protein composition, lactose levels, non-fat solids, titratable acidity, and freezing point depression. These metrics not only characterize the compositional profile of dairy products but also exhibit significant correlations with ethanol stability. Numerous studies have demonstrated that factors influencing ethanol stability in milk manifest multidimensional and hierarchical characteristics. Chavez et al. [11] revealed that ethanol-unstable milk samples typically exhibit reduced titratable acidity, somatic cell counts, casein content, and non-fat solids, concomitant with elevated freezing points and higher concentrations of chloride ions and sodium-potassium ions. Meanwhile, Fagnani et al. [12] investigated the impact of lactose crystal morphology on ethanol stability, identifying a predominance of α-lactose crystals in unstable milk compared to alternative crystalline forms in stable counterparts. Horne et al. [13] systematically reviewed the evolution of ethanol stability research, highlighting its original application as a freshness indicator for raw milk and its recent expansion as an early-warning metric for thermal processing efficacy in high-temperature treatments.

Despite the established correlations between physicochemical parameters and ethanol stability, several critical gaps remain in the current body of research. First, existing studies predominantly rely on small-scale experimental datasets or controlled laboratory conditions, limiting their applicability to large-scale industrial scenarios where data volumes are substantial and class distributions are severely imbalanced. Second, while traditional machine learning methods have been explored for dairy quality prediction, they often struggle with the nonlinear complexities inherent in multi-parameter interactions and fail to provide interpretable feature selection mechanisms essential for industrial adoption. Third, the extreme scarcity of ethanol-unstable samples in real production environments—a consequence of stringent upstream quality control—poses a fundamental challenge that has not been adequately addressed through conventional data balancing techniques. These limitations collectively hinder the development of reliable, non-destructive, and industrially deployable soft sensing systems for ethanol stability assessment.

To address these gaps, this study makes three principal contributions. First, from a methodological perspective, we introduce a novel integration of TabNet’s [14] instance-wise attention mechanism with TabDDPM-based diffusion modeling [15], creating a framework specifically designed for extreme class imbalance in tabular industrial data. Unlike conventional oversampling methods that risk generating unrealistic samples, our diffusion-based approach learns the underlying data distribution to produce physically plausible synthetic samples while respecting domain constraints. Second, from a practical engineering perspective, we demonstrate that ethanol stability can be predicted with 92.57% accuracy and 90.26% recall using only routine physicochemical measurements already collected during milk intake, thereby eliminating the need for destructive testing and enabling real-time quality monitoring at industrial scale. Third, from an application-oriented perspective, we validate our model on 907,595 real-world records spanning three years of production data, providing empirical evidence that deep learning-based soft sensing can operate reliably under the extreme imbalance ratios (1:63) characteristic of actual dairy processing environments. These contributions establish both the theoretical foundation and practical pathway for intelligent, non-destructive ethanol stability assessment in industrial dairy quality control systems.

## 2. Materials and Methods

### 2.1. Materials

A Lenovo ThinkSystem SR650 server (Lenovo, Beijing, China) was used for model training and data processing.

Acceleration was provided by an NVIDIA A100 GPU (NVIDIA Corporation, Santa Clara, CA, USA).

### 2.2. Raw Data

This study employs raw milk as the primary material, with analytical data sourced from 907,595 full-batch inspection records of raw milk intake at a dairy enterprise, spanning January 2022 to October 2024. Each registered batch documents essential metadata (e.g., order status, transaction date, inspection lot number). Key quality parameters include:

1. Acceptance criteria (rejection status, rejected volume, rejection reasons, etc.);
2. Physicochemical properties (total bacterial count, protein content, total solids, stability, etc.);
3. Veterinary drug residues (sulfonamides, metamizole, tetracyclines, etc.);
4. Pesticide residues (DDT, dieldrin, aldrin, etc.);
5. Contaminant screening (AD1, AD2, melamine, etc.);
6. Toxin monitoring (aflatoxin M1, along with other mycotoxins).

All measurements were conducted under standardized laboratory protocols at the collaborating dairy facility, ensuring regulatory compliance and traceability.

### 2.3. Data Selection

The prediction of milk ethanol stability is the goal of this study. Considering the correlation and detection rate, 15 high-frequency detection indicators related to ethanol stability were preliminarily selected in the quality evaluation of raw milk: total viable count, protein content, total solids, solids-not-fat, fat content, acidity, freezing point, somatic cell count, psychrotrophs, lactose content, relative density, milk temperature, color, AD1 and AD2, and the detection methods of each index were in Table 1.

### 2.4. Data Cleaning

The raw dataset, although extensive, required substantial preprocessing owing to missing entries and irregular formatting. The data-cleaning workflow began with a full extraction of the source database, followed by the selection of fifteen routinely measured indicators serving as initial candidate features. Subsequent steps included removal of records with null values, encoding of categorical variables (positive/negative assigned as 1/0), and standardization of numerical fields by eliminating non-numeric strings to retain syntactically valid values. After preprocessing, the dataset was restructured with ethanol stability positioned as the leading target variable while preserving the original ordering of the remaining features, and the final cleaned data were exported as a standardized CSV file for downstream analysis. The complete data-cleaning procedure is outlined in Appendix A Algorithm A1.

Following preprocessing, a total of 54,326 valid entries were retained, consisting of 842 positive samples (ethanol instability) and 53,484 negative samples (ethanol stability), forming a severely imbalanced dataset. To mitigate this imbalance, diffusion-based data augmentation was subsequently applied to enrich the minority class. Given the fifteen initial features extracted from routine measurements, their potential relevance to ethanol stability prediction—as well as possible redundancies among them—necessitated systematic evaluation. Accordingly, both linear and nonlinear correlation analyses were performed to quantify feature–target associations and assess inter-feature substitutability.

### 2.5. Autoencoder Correlation Analysis

Autoencoders, as a canonical unsupervised learning architecture, are fundamentally designed for dimensionality reduction and feature extraction. Their core mechanism enforces the minimization of reconstruction errors, thereby compelling latent encodings to capture essential data characteristics and nonlinear interdependencies among variables [24]. Common variants include Denoising Autoencoders, which enhance robustness through input noise perturbation; Sparse Autoencoders, which impose sparsity constraints to isolate discriminative features; and Variational Autoencoders, which construct probabilistic latent spaces to quantify distributional correlations. The selection of latent dimensionality represents a critical design choice in autoencoder-based feature learning. While higher dimensions can capture more nuanced data variations, they risk introducing redundancy and computational overhead; conversely, excessively low dimensions may result in information loss. To determine the optimal latent dimension for this study, we conducted systematic experiments with dimensions ranging from 2 to 5. A latent dimensionality of 3 was ultimately selected based on reconstruction error analysis and downstream classification performance. This configuration achieved the lowest reconstruction error while maintaining interpretable latent representations that clearly separated positive and negative samples in visualization. Dimensions higher than 3 yielded negligible improvements in reconstruction accuracy but introduced computational complexity, whereas dimensions lower than 3 resulted in incomplete capture of the data’s distributional structure. The three-dimensional latent space proved sufficient to encode the essential physicochemical patterns influencing ethanol stability, with each latent variable capturing distinct compositional characteristics.

### 2.6. Data Balancing Methodology

Training classification models on class-imbalanced datasets poses well-known difficulties. When positive and negative samples differ greatly in number, the resulting imbalance often produces broad regions of overlap in the feature space, making it harder for the model to form clear and reliable decision boundaries. At the same time, the limited number of positive samples tends to create sparse and fragmented feature distributions. Under such conditions, informative but infrequent patterns may be mistaken for noise and thus receive insufficient attention during model learning. The restricted coverage of positive samples also prevents the model from capturing their overall distribution, increasing the risk of overfitting to small, localized regions and ultimately weakening generalization.

Common remedies for class imbalance include interpolation-based oversampling methods such as SMOTE (Synthetic Minority Over-sampling Technique) and ADASYN (Adaptive Synthetic Sampling), which generate minority-class samples by combining existing observations in the feature space. While these approaches are often effective, they are not well suited to physicochemical milk data, where variables are linked by domain constraints and strong multivariate dependencies. For example, all compositional indicators must remain non-negative, and total solids should be consistent with the sum of fat and solids-not-fat within a reasonable tolerance. Interpolation may violate such constraints or produce unlikely combinations of correlated attributes, requiring additional filtering and reducing the practical usefulness of the generated samples. For this reason, we adopted a diffusion-based generation approach, which learns the joint distribution of the data and allows physical plausibility checks to be incorporated during sample generation.

Diffusion models [25] rely on two complementary stages: a forward process that gradually corrupts the data by adding Gaussian noise according to a predefined schedule, and a reverse process in which a neural network is trained to remove this noise step by step. By learning how to map pure noise back to the data distribution, the model can generate new samples that resemble the original data.

For tabular datasets, however, diffusion-based generation requires additional adaptations. Because such data typically combine continuous numerical features with discrete categorical ones, the forward noising mechanism for continuous variables is commonly modelled as a Markov chain. At each timestep, the following state is obtained by blending the current value with injected Gaussian noise, as expressed in Equation (1).

(1) $$x_{t}=\sqrt{\alpha_{t}}x_{t-1}+\sqrt{1-\alpha_{t}}\epsilon_{t}$$

where $\alpha_{t}=1-\beta_{t},\;\beta_{t}$ is predefined noise variance tables, $\epsilon_{t}∼N\left(0,\;I\right)$.

By accumulating parameters $\bar{\alpha }=\prod_{i=1}^{t}\alpha_{i}$, the noise data at any time step can be directly calculated from the initial $x_{0}$, which avoids the computational overhead of iterating time steps, as shown in Equation (2).

(2) $$x_{t}=\sqrt{{\bar{\alpha }}_{t}}x_{0}+\sqrt{1-{\bar{\alpha }}_{t}}$$

For categorical features, one-hot encoding is first applied to transform them into model-compatible inputs. To maintain consistency with the continuous-variable formulation, the perturbation of categorical variables is modeled using a multinomial diffusion process. At each timestep, the forward transition is given by Equation (3).

(3) $$x_{t}\sim q\left(x_{t}\right|x_{t-1})=Cat(x_{t};\left(1-\beta_{t}\right)x_{t-1}+\frac{\beta_{t}}{K}\cdot 1)$$

where K is the number of categories, and 1 is a K-dimensional uniform probability vector. This formulation introduces categorical perturbation noise by gradually flattening the one-hot distribution toward the uniform distribution, analogous to Gaussian corruption in continuous-feature diffusion.

Ensuring the technological validity and physical plausibility of synthetic samples required the imposition of two critical constraints during the diffusion-based generation process. The first constraint enforces strict positivity of all feature values, which stems from the fundamental nature of physicochemical measurements: parameters such as protein content, fat percentage, and lactose concentration represent inherently non-negative quantities, and negative values would signify physically impossible states. The second constraint addresses the compositional integrity of milk: total solids represent the combined mass of all solid components, which can be partitioned into fat and solids-not-fat. By definition, total solids should equal the sum of fat and solids-not-fat; however, minor deviations may occur due to measurement uncertainties or rounding in industrial settings. We therefore enforce that the absolute difference between total solids and the sum of fat and solids-not-fat must not exceed 0.5%, permitting realistic measurement variations while preventing compositionally invalid samples. These constraints serve dual purposes: they prevent the generation of physically nonsensical data that could mislead the downstream classifier, and they ensure that the synthetic samples maintain the technological relevance required for industrial application. Implementation was accomplished through rejection sampling during the reverse diffusion process, where generated candidates violating either constraint were discarded and regenerated.

In the reverse generation phase, the model architecture employs a multilayer perceptron (MLP), a structure widely adopted for tabular data, to predict the noise added at each diffusion timestep [26]. This design leverages the MLP’s capacity to capture nonlinear mappings between perturbed categorical features and their denoised counterparts while remaining computationally efficient for structured data.

### 2.7. Tabnet-Based Soft Sensing Model

TabNet, developed by Google Research for tabular data, combines the interpretability of tree-based methods with the flexibility of deep neural networks [27]. A central feature of the architecture is its instance-wise feature selection: at each decision step, the model determines which features are most relevant for the current sample. Sparsemax is used to form the attention masks, encouraging the model to focus on only a small subset of inputs and helping maintain interpretability without sacrificing modelling capacity.

**Figure 1:**
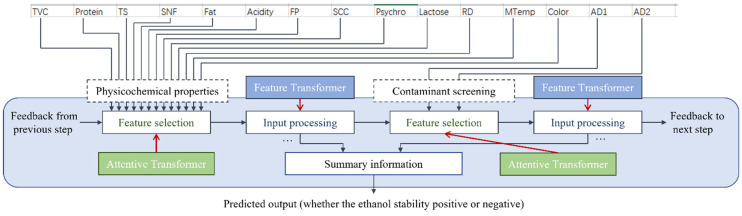
The structure of TabNet. Arrows indicate the flow of information and feedback between different modules.

TabNet does not follow a traditional encoder–decoder design. Instead, it operates through a sequence of decision steps. Each step contains two modules: a Feature Transformer that applies both shared and step-specific nonlinear transformations, and an Attentive Transformer that produces feature masks to guide which inputs will be used in the following step. The outputs from all decision steps are then aggregated to form the final prediction. This sequential structure allows the model to learn expressive representations while still making its feature-selection logic transparent. Figure 1 illustrates the overall structure.

These characteristics make TabNet particularly suitable for industrial soft sensing applications. Unlike conventional tree-based ensemble models, which typically provide global or post-hoc feature importance measures, TabNet enables instance-wise interpretation by explicitly quantifying the contribution of each input feature for individual samples. This capability is especially valuable in quality screening scenarios, where understanding why a specific batch is classified as potentially unstable is critical for operator trust and subsequent decision-making.

Using these characteristics of TabNet, this study constructs a soft-sensing model to predict the alcohol stability of raw milk. The model takes routinely measured quality indicators as inputs and estimates alcohol stability without the destructive procedures required by laboratory assays. By learning the nonlinear relationships between measured variables and the target property, the method provides a practical alternative to conventional testing. It also offers a basis for real-time quality monitoring and early detection of potential issues in raw-milk processing.

### 2.8. Experimental Setup for Data Generation and Result Prediction

In both the data generation and alcohol stability soft sensing model development stages, all experiments were conducted on a Lenovo SR650 server equipped with an NVIDIA A100 GPU, with model implementation and training carried out using the PyTorch 2.0.1 framework.

During the data generation phase, a base dataset comprising 842 real positive samples and 3368 randomly sampled negative samples was constructed. A total of 4210 positive samples were generated using a diffusion model. To ensure the physical plausibility of the generated data, two physical constraints were introduced: (1) all selected feature values must remain strictly positive, and (2) the absolute deviation between the total solids content and the sum of fat and solids-not-fat must be within 0.5 units. The diffusion model was trained for 1000 epochs with a batch size of 256, using the AdamW optimizer and a dynamic learning rate schedule, with the initial learning rate set to 0.001.

Based on the generated samples, two datasets were constructed for model training and evaluation. The real dataset consisted of 842 positive and 3368 negative samples. The augmented dataset comprised 5052 positive samples (including 3368 generated samples, 842 original samples, and 842 duplicated original samples) together with 15,156 negative samples. Prior to model training, hyperparameter optimization was performed using the Optuna framework. The search space covered key parameters including the embedding dimension for input features (8–64), attention dimension (8–64), number of decision steps (3–10), regularization coefficient (10^−3^–10^−1^), sparsity regularization coefficient (10^−5^–10^−1^), learning rate (10^−5^–10^−1^), and batch size (16–128). While the number of raw input variables remained fixed at 15, the embedding dimension served as a model-internal parameter governing the size of each feature’s latent representation. The final soft sensing model was evaluated in comparison with multilayer perceptron (MLP) and gradient-boosted decision tree (GBDT) [28] models.

In practical industrial dairy production, raw-milk quality data are continuously accumulated and reused over time, and strict sample-level separation between historical training data and evaluation data is often difficult due to repeated sampling, batch overlap, and reuse of routine measurements. Accordingly, model evaluation in this study was conducted under a production-like setting in which the training and evaluation datasets were not strictly disjointed at the sample level. The reported results should therefore be interpreted as an estimate of screening performance under realistic industrial data reuse conditions, rather than as a strict measure of out-of-sample generalization.

Regarding the required sample size for applying the proposed soft sensing software, the method does not depend on a strict minimum number of samples in principle. Instead, its performance is primarily influenced by the representativeness and balance of the available data. Based on empirical observations during model development and validation, a dataset containing several hundred ethanol-unstable samples together with a few thousand ethanol-stable samples is sufficient to train a stable and reliable prediction model.

In the present study, the original dataset included 842 ethanol-unstable samples, which already enabled effective model training and evaluation. To further enhance robustness under severe class imbalance commonly encountered in industrial scenarios, a diffusion-based data augmentation strategy was introduced to enrich the minority class. As a result, the developed software is well suited for industrial applications where long-term routine inspection data are accumulated, and its predictive performance can be continuously improved as additional data become available.

## 3. Results

To explicitly demonstrate both the experimental data and the results of computer-based processing, this section presents a series of quantitative and visual analyses. The distributions of original physicochemical measurements and diffusion-generated samples are compared to illustrate data-level processing effects. Furthermore, classification results, confusion matrices, and performance metrics are reported to demonstrate the outcomes of computer-based prediction.

### 3.1. Data Correlation Analysis

This study simultaneously employs explicit feature relevance analysis, including interpretable indicators such as ANOVA F-scores [29], Point-biserial correlation [30], Random Forest feature importance [31], and Logistic regression coefficients [32], to directly assess the relationship between features and the target variable, as well as implicit feature learning through an unsupervised autoencoder for the discovery of latent features. The former offers clear statistical or model-driven interpretability, while the latter does not depend on the target variable and instead focuses on the distributional patterns inherent in the data. The experimental dataset consists of 842 positive samples, along with 3368 randomly sampled negative samples.

**Figure 2:**
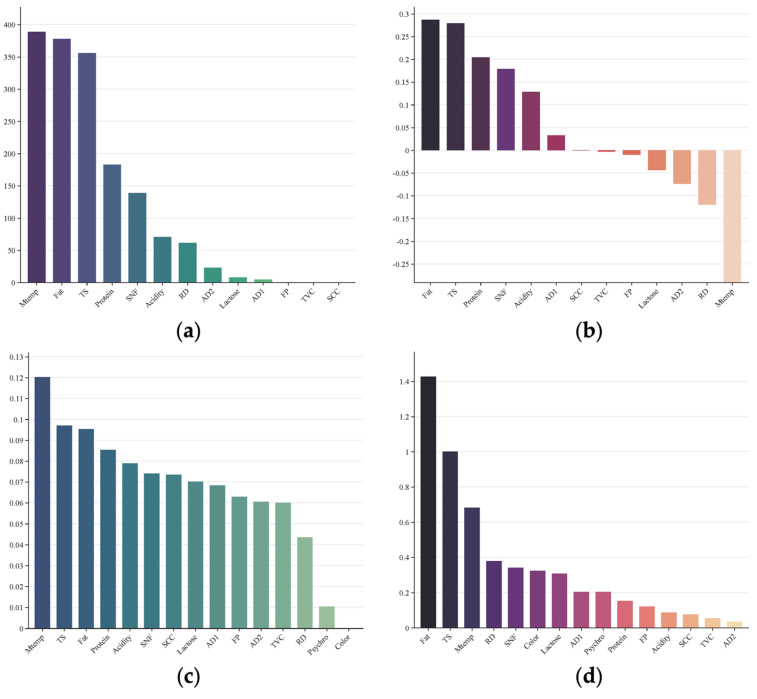
Data correlation analysis. (**a**) ANOVA f−scores for continuous features; (**b**) Point−biserial correlation; (**c**) Random forest feature importance; (**d**) Logistic regression coefficients.

Figure 2 shows the results of the explicit feature relevance analysis. Variables such as SCC, TVC, FP, AD1, AD2, Color, and Psychro exhibit weak associations, indicating that their variations are not strongly related to ethanol stability. In contrast, the remaining features, namely Protein, TS, SNF, Fat, Acidity, Lactose, RD, and MTemp, demonstrate stronger correlations with ethanol stability. These results are consistent with the findings of Chavez et al. [10] who reported that acidity significantly influences ethanol stability.

**Figure 3:**
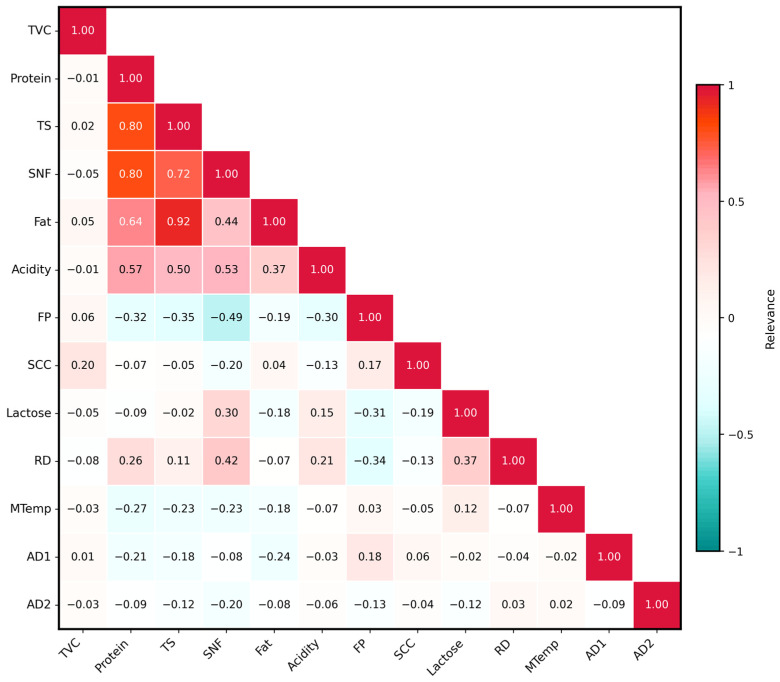
Heatmap of the relationships between features.

Figure 3 presents a heatmap illustrating the inter-feature correlations. A strong positive correlation is observed between Fat and TS, with a coefficient of 0.92. Other relatively strong correlations are primarily found among the four features: TS, SNF, Fat, and Protein.

**Figure 4:**
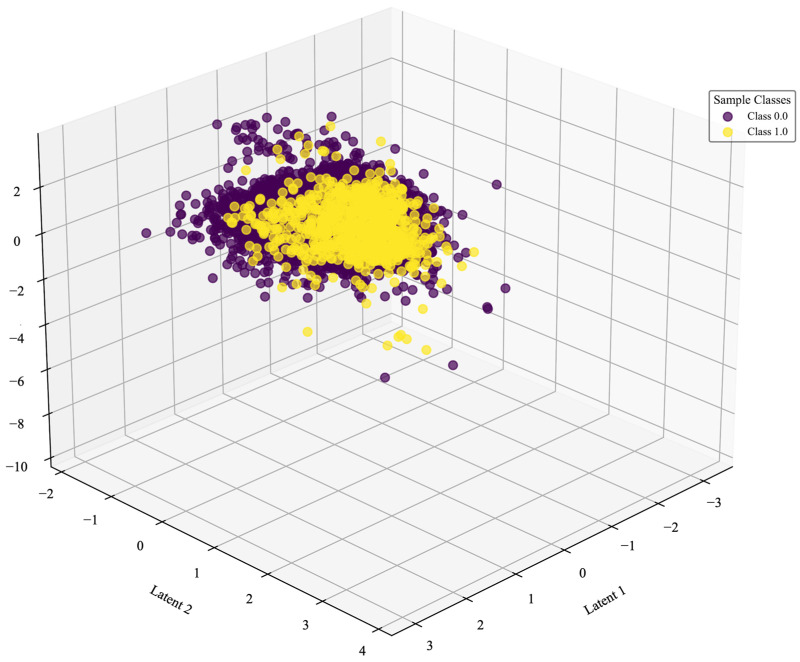
Autoencoder 3D latent space distribution.

Figure 4 presents the results of the autoencoder-based implicit feature learning. Three latent variables were selected for analysis and visualized in a three-dimensional latent space. The positive and negative samples exhibit relatively compact clustering, though a small number of outliers are observed—particularly in the positive direction of Latent 2. A relatively clear separation is evident between the positive and negative samples, with the positive samples showing a more compact distribution. This suggests a certain degree of structural similarity among the positive samples, making them more easily compressed into concentrated latent representations. In contrast, the broader distribution of negative samples implies greater variability or the presence of more noise in their features. Notably, the most pronounced separation between the two classes occurs along Latent 2.

**Figure 5:**
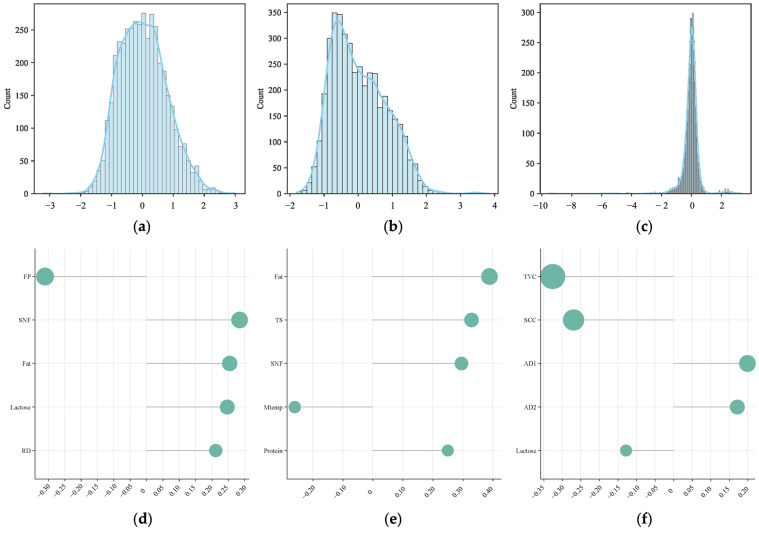
Distribution and composition of latent variables. (**a**) Latent 1 distribution; (**b**) Latent 2 distribution; (**c**) Latent 3 distribution; (**d**) Latent 1 composition; (**e**) Latent 2 composition; (**f**) Latent 3 composition.

Figure 5 illustrates the distribution and feature composition of each latent variable. As shown in subplots Figure 5a–c, Latent 1 approximates a normal distribution with slight right skewness; the peak is centered near 0 with a slightly extended right tail, suggesting the occasional presence of high encoding values. This indicates that Latent 1 has learned relatively symmetric features, with a small number of strong features or outliers contributing to the skew. Latent 2 exhibits a clearly right-skewed distribution, with a mean near −0.5 and a pronounced long tail extending to values around 3 to 4. In addition to the overall skewness, Latent 2 shows signs of minor multimodality, where small additional peaks appear along the density curve, suggesting that this latent dimension may be capturing several weakly separated structural patterns within the data. This may reflect a rare pattern or anomalous structure captured in the data. Latent 3 is sharply peaked, resembling a Laplace-like distribution, with a strong concentration around 0 and long tails on both sides, particularly on the left, reaching as low as −10. This implies that most samples have encoding values close to 0 in this dimension, while a few are encoded with extreme values, suggesting potential utility in anomaly detection. Latent 3 may represent a compact encoding of “abnormality”.

As shown in subplots Figure 5d–f, Latent 1 is primarily composed of FP, SNF, Fat, Lactose, and RD; Latent 2 is mainly composed of Fat, TS, SNF, MTemp, and Protein; and Latent 3 is largely composed of TVC, SCC, AD1, AD2, and Lactose. Among them, Latent 2, composed of Fat, TS, SNF, MTemp, and Protein, emerges as the most significant latent variable.

Based on both types of feature relevance analysis, the final selected features include Protein, TS, SNF, Fat, Acidity, Lactose, RD, and MTemp.

### 3.2. Results of the Diffusion Model-Generated Data

**Figure 6:**
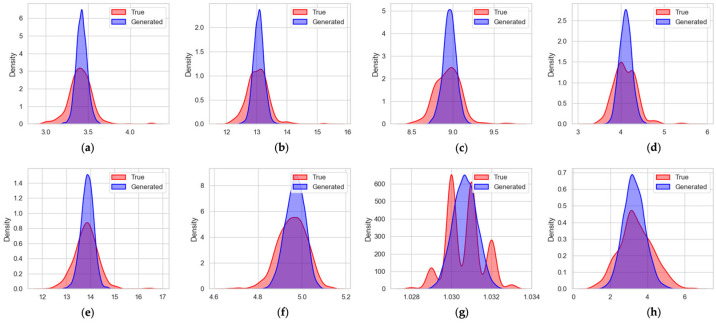
Distribution of generated data and original data. (**a**) Distribution of protein; (**b**) Distribution of total solid; (**c**) Distribution of solids not fat; (**d**) Distribution of fat; (**e**) Distribution of acidity; (**f**) Distribution of lactose; (**g**) Distribution of relative density; (**h**) Distribution of milk temperature.

A total of 3368 positive samples with complete feature information were generated by the diffusion model, covering eight key indicators: Protein, TS, SNF, Fat, Acidity, Lactose, RD, and MTemp. The quality evaluation of the generated data was based primarily on distributional similarity, with detailed comparisons illustrated in Figure 6.

The visual comparison in Figure 6 indicates that the diffusion model achieves a generally strong performance in reconstructing the marginal distributions of the eight physicochemical attributes. For protein, acidity, lactose, and milk temperature, the alignment between the generated and true distributions is particularly noteworthy. The synthetic density curves closely coincide with the empirical ones, showing well-matched modes, similar dispersion, and substantial overlap across the entire support. These results demonstrate that the model captures the essential statistical characteristics of these variables, including both central tendency and overall distributional shape, with high fidelity.

For the remaining features—total solids, solids not fat, fat, and relative density—the generated data still reflect the general structure of the original distributions, though with some degree of deviation. In total solids, solids not fat, and fat, the synthetic curves display slightly higher central values and a mild rightward shift; however, the model successfully preserves the overall unimodal tendency and maintains a comparable spread. Although the true distributions of these variables exhibit subtle multimodal fluctuations, the generated data provide a smooth approximation that captures the principal mass and dominant peak of the distributions.

Relative density presents the most complex distribution among the eight attributes, characterized by clear multimodality in the real data. Although the diffusion-generated samples reproduce the overall range of relative density values, noticeable discrepancies remain between the generated and empirical distributions, particularly in terms of shape and local density. This indicates that the diffusion model does not fully capture the true distribution of relative density in this case. Such limitations may introduce uncertainty in the learned contribution of relative density to ethanol stability prediction. However, since relative density represents only one of multiple correlated physicochemical indicators used by the model, its imperfect reconstruction does not dominate the overall prediction performance. Improving the fidelity of generated distributions for weakly varying features such as relative density remains an important direction for future work. This suggests that the model can approximate complex distributional structures even when the underlying pattern is highly irregular.

Overall, the diffusion model demonstrates robust generative capability, accurately reconstructing the distributions of several key attributes and providing reasonable approximations for variables with more intricate statistical patterns. In view of these observed differences, an additional copy of the real positive samples was incorporated into the final training dataset, resulting in each original positive sample appearing twice. This deliberate duplication was designed to reinforce the influence of the true data distribution and enhance training stability when integrating synthetic and real samples.

### 3.3. Classification Model Experimental Results

The soft sensing model evaluation was conducted using both a standard test set and a complete production dataset. Here, the “test dataset” refers to the held-out test portion of the training dataset used during model development, whereas the “all dataset” corresponds to the full three-year raw-milk intake dataset provided by the dairy enterprise, containing all available real-world samples. The model was initially trained on a predefined training and test split, after which the fully trained model was deployed for validation on the full-scale production dataset. The optimal hyperparameter configuration for TabNet, obtained via hyperparameter tuning, was as follows: input dimension of 62, attention embedding dimension of 20, number of decision steps set to 3, regularization coefficient of 0.038, sparsity regularization coefficient of 0.026, learning rate of 0.012, and batch size of 58. The final soft sensing model was trained based on this configuration.

**Figure 7:**
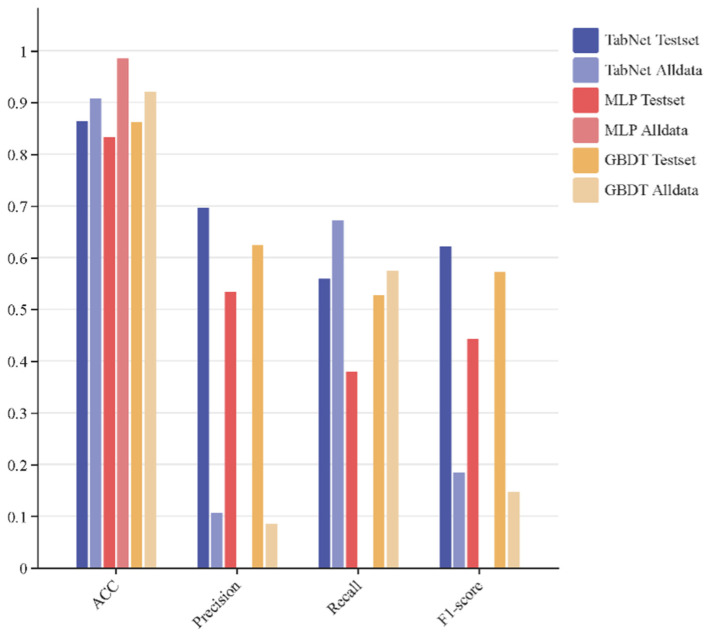
Results of soft sensing models without data generation.

Figure 7 compares the performance of TabNet, MLP, and GBDT on both the test set and the production dataset, evaluated in terms of accuracy, precision, recall, and F1-score. Experimental results indicate that all models perform significantly better on the test set than on the production data. This performance gap arises because the production dataset represents the dairy company’s entire three-year intake record, containing an extremely low ratio of positive samples, where even a 10% false positive rate among negative samples can cause false positives to far exceed the total number of true positives. Consequently, all models exhibit poor precision when applied to the production dataset. In contrast, recall scores are relatively more robust, with all models except MLP achieving values above 0.55. Notably, MLP yields zero values for precision, recall, and F1-score on the production dataset; its superficially high accuracy results from a degenerate decision strategy that classifies all instances as negative.

A critical distinction must be drawn between model performance on the held-out test set versus the full production dataset, as these two evaluation scenarios present fundamentally different challenges. The test set, derived from the curated training data, contains a balanced representation of positive samples with approximately 20% positive rate, enabling standard evaluation metrics to furnish meaningful performance assessment. In this controlled setting, all three models achieve strong performance, with TabNet demonstrating precision and recall values exceeding 0.85.

The production dataset, in stark contrast, reflects the reality of industrial dairy operations, where stringent upstream quality control results in an extremely low prevalence of ethanol-unstable milk at approximately 0.09% positive rate, corresponding to a 1:1100 imbalance ratio. Under such extreme imbalance, conventional accuracy metrics become misleading: even a naive classifier that labels all samples as negative would achieve accuracy exceeding 99%. More critically, a false positive rate of merely 0.1% among the overwhelming majority of negative samples generates a number of false alarms far exceeding the total number of true positives, causing precision to collapse despite maintained recall. This phenomenon accounts for why all models exhibit precision values below 0.05 on the production dataset while maintaining recall above 0.55, with the exception of MLP, which degenerates to a trivial all-negative classifier. For industrial deployment, recall emerges as the paramount metric, as it quantifies the model’s capacity to detect genuinely unstable milk and prevent quality failures downstream.

**Figure 8:**
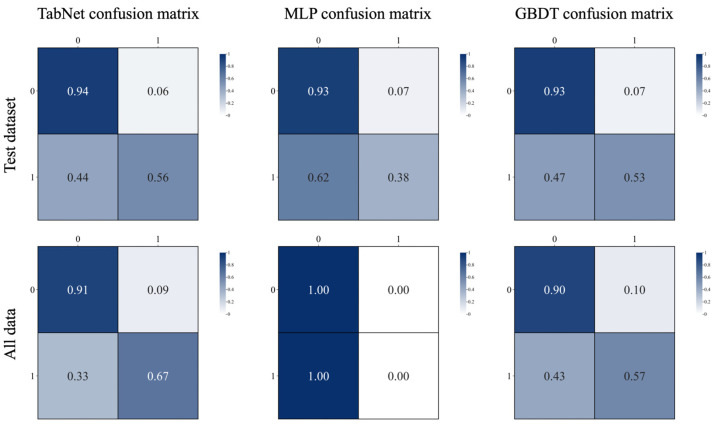
Confusion matrix of soft sensing model without data generation.

Figure 8 further presents confusion matrices for both datasets, providing a clear visualization of MLP’s failure mode. A side-by-side comparison of TabNet and GBDT reveals that TabNet achieves a slight performance advantage. This performance gap is attributable to the sensitivity of gradient boosting decision trees to noisy outliers among the limited number of positive samples, which hinders the model’s ability to realize its full potential. Based on comprehensive performance evaluation, TabNet was ultimately selected as the classification backbone for the proposed soft sensing model.

In this context, GBDT is used as a representative tree-based ensemble method, conceptually aligned with widely adopted implementations such as XGBoost and LightGBM. While such models often provide strong baseline performance on tabular data, their decision logic is typically interpreted using global or post-hoc feature importance measures. By contrast, TabNet offers instance-wise feature attribution through its learned attention masks, enabling a more transparent examination of how individual physicochemical indicators contribute to specific predictions. This property is particularly advantageous for industrial quality screening applications, where understanding the rationale behind batch-level decisions is essential.

**Figure 9:**
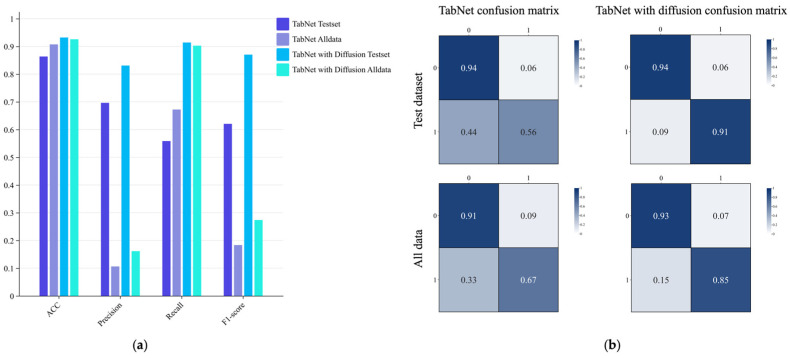
Comparison results of soft sensing models with and without using generated data. (**a**) Bar chart of prediction results; (**b**) Prediction confusion matrix.

Building on the selection of TabNet as the core classifier, subsequent experiments focused on assessing the impact of diffusion-based data augmentation. Specifically, model performance was compared with and without the inclusion of synthetic positive samples during training. As illustrated in Figure 9, the impact of the data augmentation strategy on model performance is evident. When the number of positive samples was increased to 5052 through the inclusion of generated data, the model exhibited a marked reduction in false-negative predictions. The soft sensing model trained with augmented data demonstrated significant improvements in both accuracy and recall. Although precision and F1-score also showed observable gains relative to the baseline model, further improvement remains possible due to the inherently extreme imbalance between positive and negative samples in the original dataset. Nevertheless, the achieved accuracy of 92.57% and recall of 90.26% provide compelling empirical evidence that the proposed soft sensing model for ethanol stability in raw milk holds substantial potential for practical engineering applications.

To assess the stability of the proposed soft sensing model, repeated training experiments were conducted under varying conditions. Five independent training runs were performed using the optimal hyperparameters identified through Optuna optimization, specifically n_d = 62, n_a = 20, n_steps = 3, gamma = 0.038, lambda_sparse = 0.026, learning_rate = 0.012, and batch_size = 58, but with different random initializations. These runs yielded an accuracy range of 91.31–92.57%, alongside a recall range of 88.93–90.26% on the test set. These experimental results demonstrate that model performance remains robust to initialization and does not depend on fortuitous random seeds.

Hyperparameter sensitivity analysis further revealed that the model maintains stable performance when parameters deviate from their optimal values within reasonable ranges: accuracy varied by less than 1.5% when the learning rate was adjusted between 0.008 and 0.016 around the optimal value of 0.012, and by less than 2% when the number of decision steps ranged from 2 to 5 compared to the optimal value of 3. The regularization coefficients showed minimal impact on recall with less than 1% variation when varied around their optimal values of gamma = 0.038 and lambda_sparse = 0.026, confirming that the model’s core capability to identify positive samples does not critically depend on fine-tuned regularization. These results indicate that the proposed approach can be reliably deployed without requiring extensive hyperparameter retuning when adapted to new dairy facilities or updated production data, thereby enhancing its practical applicability for industrial quality control systems.

## 4. Discussion

This study proposes a TabNet-based soft sensing approach for evaluating ethanol stability in raw milk. By leveraging routinely collected physicochemical measurements, the model offers a practical alternative to laboratory ethanol tests, which are inherently destructive and time-consuming. The use of sequential attention within TabNet allows the model to focus on the most informative variables at each decision step, helping explain the basis of its predictions and making the method suitable for industrial environments that require traceable decision processes.

Despite the strong performance observed in this study, several limitations should be acknowledged. The model was developed using data from a single dairy enterprise, and its performance may therefore depend on specific regional conditions, feed management practices, and processing standards. Variations in cow breed, seasonal feeding patterns, climate, and herd management across different regions may influence milk composition and ethanol stability, indicating that cross-enterprise validation and potential retraining are necessary to ensure broader generalizability.

The three-year dataset provides substantial coverage of routine industrial operations but may not fully reflect long-term structural changes such as gradual shifts in feeding strategies, selective breeding effects, or modifications in milk collection logistics. These factors can introduce distributional changes over time, highlighting the need for periodic model updating during long-term deployment.

In addition, the present work focuses on ethanol stability prediction rather than on identifying the underlying causal mechanisms of instability. While the model effectively flags high-risk batches, it does not provide direct recommendations for corrective actions, such as adjustments to feed formulation or processing parameters. Extending the framework toward causal or explanatory analysis could further enhance its value as a decision-support tool.

The extreme class imbalance observed in this dataset likely reflects a well-managed industrial setting in which ethanol-unstable milk is rare. Production environments with higher rejection rates may require recalibration of decision thresholds or alternative balancing strategies to maintain reliable performance.

Recent advances in intelligent sensing and data-driven dairy quality assessment support the feasibility of the proposed approach. Studies combining electronic-nose systems with machine-learning algorithms [33] and AI-enabled IoT sensors have demonstrated the potential of virtual sensing for continuous milk quality monitoring [34]. In this context, the present study further shows that large-scale industrial datasets, despite inherent noise and imbalance, contain exploitable structure that can be effectively learned using appropriate modeling and data augmentation strategies [35]. Diffusion-based data generation improved the representation of rare ethanol-unstable samples and contributed to more robust decision boundaries, consistent with findings in other food-quality prediction tasks [36].

From an application perspective, the proposed soft sensing model has clear industrial relevance: it relies on measurements that are already routinely collected, can be executed in real time, and supports early identification of instability risks before raw-milk storage [37,38]. These characteristics are consistent with ongoing efforts toward more automated, sensor-supported HACCP systems in dairy production, highlighting the broader movement toward digital and intelligent quality-management frameworks [39].

Beyond overall performance metrics, the practical consequences of prediction errors must be considered from an industrial risk-management perspective. The costs associated with false positives and false negatives are highly asymmetric in ethanol stability screening. A false negative, in which ethanol-unstable milk is incorrectly classified as stable, may allow unsuitable raw milk to enter downstream processing, increasing the risk of protein coagulation, product quality defects, and economic loss. In contrast, a false positive primarily results in additional confirmatory laboratory testing or temporary batch holding, which incurs relatively limited operational cost. For this reason, the proposed soft sensing model is intentionally optimized to prioritize recall, ensuring that potentially unstable batches are rarely missed. This design choice is consistent with conservative quality-control practices in dairy processing, where risk mitigation is favored over maximal throughput.

It should be emphasized that the proposed method provides a probabilistic classification rather than a direct physicochemical measurement. As a result, occasional false-positive or false-negative predictions are unavoidable. The model is therefore intended to complement, rather than replace, conventional ethanol stability testing by serving as an early-warning and screening tool within industrial quality-control workflows.

In practical implementation, routine physicochemical measurements can be automatically transmitted to the soft sensing system through existing laboratory information or process control infrastructure. Batches flagged as potentially unstable can then be prioritized for confirmatory laboratory testing, improving operational efficiency while reducing unnecessary destructive analyses.

Looking ahead, integrating the proposed soft sensing approach with actual in-line sensor systems represents a promising direction. Research in Sensors has shown that coupling AI models with real-time sensing hardware—such as spectroscopic probes, flow-temperature monitors, or IoT sensor arrays—can significantly strengthen prediction reliability under varying processing conditions [40]. Future work should also evaluate model generalizability across different regions, seasons, and milk sources, as well as extend the soft sensing framework to additional quality indicators to support more comprehensive decision-making in dairy operations [41].

## 5. Conclusions

This study developed a TabNet-based soft sensing model for estimating ethanol stability in raw milk using routine physicochemical measurements. By applying latent-feature reconstruction, correlation-based feature evaluation and diffusion-model augmentation to compensate for the scarcity of ethanol-unstable samples, eight effective predictors were identified. Using these inputs, the model reached 92.57% accuracy and 90.26% recall on a three-year industrial dataset, showing that ethanol stability can be inferred reliably from non-destructive measurements already collected during milk intake.

The results demonstrate that TabNet can extract meaningful structure from large, imbalanced raw-milk datasets and provide a practical substitute for traditional ethanol tests. Because the approach relies solely on existing operational data, it offers a straightforward path for supporting routine quality assessment in dairy processing environments.

Future research will focus on validating the proposed framework across multiple dairy enterprises to assess its generalizability, as well as extending the soft sensing approach to additional milk quality indicators. These efforts will further enhance the applicability of data-driven soft sensing methods for intelligent dairy quality control.

## Figures and Tables

**Table 1:** Detection methods of main indicators.

Indicators	Standards	Steps	Sensor Principle	Implementation
Ethanol Stability (ES)	GB/T 6914-1986 [16]	Mix equal volumes of milk and ethanol solution at room temperature, allow to stand for 5 min, then observe for turbidity, flocculation, or coagulated masses.	Visual coagulation/protein precipitation	Off-line
Total Viable Count (TVC)	ISO 4833-1:2013 [17]	Homogenize 1 mL of a serially diluted sample with 15–20 mL of molten PCA (pH 7.0 ± 0.2), pour plate, incubate at 30 °C for 72 h, count colonies (10–300 range), and calculate CFU/mL.	Culture-based microbial growth	Off-line
Protein Content	ISO 9622:2013|IDF 141:2013	Milk is pumped through a mid-IR flow cell where absorbance at specific wavelengths is analyzed and converted into protein content using a calibration model.	Mid-infrared absorption spectroscopy	On-line
Total Solids (TS)	ISO 6731:2010|IDF 21:2010 [18]	Dry a pre-weighed milk sample in a flat-bottom dish at 102 ± 2 °C to constant mass, cool in desiccator, and calculate residue percentage.	Gravimetric mass loss	Off-line
Fat Content	ISO 9622:2013|IDF 141:2013	Milk is analyzed via mid-IR absorbance at lipid-specific wavelengths using a model calibrated to Gerber or Babcock results.	Mid-infrared absorption spectroscopy	On-line
Solids-Not-Fat (SNF)	Reference measurements	Calculated by subtracting the fat content from total solids.	Calculation	On-line
Titratable Acidity	ISO 6091:2010|IDF 86:2010 [19]	Titrate milk with 0.1 mol/L NaOH to pH 8.4 using phenolphthalein and Co^2+^ indicator, express acidity in mL NaOH/10 g SNF or convert to °Thorn.	Acid–base titration	Off-line
Freezing Point (FP)	ISO 5764:2009|IDF 108:2009 [20]	Analyze 1 mL milk sample in cryoscope, supercool, initiate crystallization, and read the freezing point from the thermal plateau.	Cryoscopy	On-line
Somatic Cell Count (SCC)	ISO 13366-1:2008|IDF 148-1:2008 [21]	Dilute and stain milk, smear onto a slide, air-dry, and count cells microscopically at 200–400×.	Optical/fluorescence cell counting	Off-line
Psychrotrophs	ISO 17410:2019 [22]	Surface-plate serial dilutions on PCA, incubate aerobically at 6.5 °C for 10 days, and count CFU.	Culture-based microbial growth	Off-line
Lactose Content	ISO 9622:2013|IDF 141:2013	Measured via mid-IR spectroscopy using absorbance at carbohydrate bands calibrated against HPLC.	Mid-infrared absorption spectroscopy	On-line
Relative Density (RD)	ISO 9622:2013|IDF 141:2013	Calculated using mid-IR spectra and optional conductivity data in a model traceable to ISO 12185 [23].	Spectral density estimation	On-line
Milk Temperature (MTemp)	Standard from Dairy Enterprise	Measured using temperature sensors.	Thermistor/RTD	On-line
Color	Standard from Dairy Enterprise	Detected using vision sensors.	Machine vision/RGB analysis	On-line
AD1, AD2	Standard from Dairy Enterprise	AD1 and AD2 are private standards of dairy company and involve pollutant detection.	Proprietary sensor systems	On-line

## Data Availability

The data used in this study were obtained from an industrial partner under a confidentiality agreement. Due to contractual and commercial restrictions, these datasets cannot be made publicly available. No new publicly shareable data were generated in this work.

## Appendix A

This is the pseudocode for a data cleaning algorithm. It illustrates the main data cleaning process.

**Algorithm A1:** Enhanced Raw Milk Data Cleaning Protocol
